# Biomechanical comparison of suspensory traction and axial traction in preoperative correction of cervical kyphosis: a finite element study

**DOI:** 10.3389/fbioe.2025.1594207

**Published:** 2025-09-01

**Authors:** Hongyu Chen, Tianchi Wu, Shengfa Pan, Li Zhang, Yanbin Zhao, Xin Chen, Yu Sun, William W. Lu, Feifei Zhou

**Affiliations:** ^1^ Department of Orthopaedics, Peking University Third Hospital, Beijing, China; ^2^ Engineering Research Center of Bone and Joint Precision Medicine, Beijing, China; ^3^ Beijing Key Laboratory of Spinal Disease Research, Beijing, China; ^4^ Department of Orthopaedics and Traumatology, The University of Hong Kong, Hong Kong, Hong Kong SAR, China; ^5^ Shenzhen Institutes of Advanced Technology, Chinese Academy of Science, Shenzhen, China

**Keywords:** cervical kyphosis, axial traction, suspensory traction, finite element, preoperative traction

## Abstract

**Objective:**

To compare the biomechanical characteristics of axial traction and suspensory traction in the process of preoperative correction of cervical kyphosis.

**Methods:**

An intact three-dimensional finite element digital model of C2-T2 with cervical kyphosis was established. The head gravity and moment were applied to the finite element model to simulate the force of skull traction and the force of suspensory traction. The changes of cervical kyphotic angle, the length of cervical spinal canal and the stress distribution of each vertebral body were analyzed under two traction modes.

**Results:**

The kyphotic angles of the kyphotic segments were reduced by both tractions. The C2-C5 kyphotic angle was 41° before traction, and decreased to 32° and 26° after axial traction and suspensory traction, respectively. The length of C3-C7 cervical spinal canal was 61.3 mm before traction. After axial traction, the length of C3-C7 cervical spinal canal increased to 61.8 mm; after suspensory traction, it decreased to 59. 6 mm. The high stress area of each vertebral body was located in the anterior longitudinal ligament attachment area of the vertebral body during both two kinds of traction. The maximum Mises stress of C2-C7 vertebral body in suspensory traction is generally small relative to axial traction.

**Conclusion:**

Compared with axial traction, suspensory traction has better kyphotic corrective effect, while reduces the length of the cervical spinal canal and the stress on the cervical vertebral body, which decreases the possibility of nerve damage and iatrogenic fracture during traction from a biomechanical point of view.

## 1 Introduction

The correction of cervical kyphosis has always been a difficult problem in spinal surgery, especially in the treatment of severe cervical kyphosis (Horn et al., 2019; Kawabata et al., 2013; Zhang et al., 2010). Direct surgical correction often fails to achieve the desired corrective effect, and there are many risks in the surgical process. Therefore, cervical kyphosis deformity often adopts a two-step strategy in the long-term clinical practice. First, through continuous neck traction, the deformity can be improved after a period of time, but also brings convenience and risk reduction for the second step of surgery (Mehrpour et al., 2017; Han et al., 2016; Horsley et al., 1997; Steinmetz et al., 2007). Preoperative traction combined with surgical correction and internal fixation has achieved good clinical results, and has been widely recognized in the treatment of severe cervical kyphosis (Kawabata et al., 2013).

Axial traction, which includes skull traction and Halo traction, is the most widely used traction method at present (Verhofste et al., 2019; Shen et al., 2019; Steinmetz et al., 2007). However it also has obvious problems, including nerve damage and iatrogenic fracture during traction (Lim et al., 2020; Pinches et al., 2004). In order to reduce the occurrence of the above complications, we designed a new traction mode, which applies force to the cervical spine from the vertical direction and was named suspensory traction. It has been proved that it can make patients have satisfactory results through clinical practice (Wu et al., 2012). In the present study, the biomechanical characteristics of traditional axial traction and suspensory traction in the process of preoperative correction of cervical kyphosis were compared by finite element analysis to provide reference for clinical practice.

## 2 Methods

### 2.1 FE modeling

A nonlinear three-dimensional finite element (FE) model of C2–T2 segments was developed and validated in our previous study (Wu et al., 2022). The model was constructed based on computed tomography (CT) images from a young male volunteer with cervical kyphosis (12 years, 168 cm, 60 kg). The CT images were obtained from image database provided by Peking University Third Hospital. All methods were carried out in accordance with relevant guidelines and regulations. The study was approved by Peking University Third Hospital Medical Science Research Ethics Committee. A statement of informed consent was obtained from the volunteer’s parents, as the volunteer was a minor at the time of the study. The CT images were put into 3D slicer (http://www.slicer.org) for geometric reconstruction of the vertebral bodies. Finite element meshing was performed using Hypermesh 2020 (Altair Technologies, Inc., CA, USA). Finally, the boundary conditions of the prepared model were set using Abaqus 2017 (Abaqus, Inc., Providence, RI, USA).

The strain on the vertebral body caused by the load applied to the model is relatively small, so cortical bone was not distinguished from cancellous bone during vertebral body construction, which was intended to simplify the calculation process. The biomechanical behavior of bone was evaluated through a phantom-less bone mineral density (BMD) measurement (Table 1) (Liu et al., 2022). Then, BMD was converted to Young’s modulus.

**TABLE 1:** Individual material properties in the finite element model.

Component	Young’s modulus (MPa)	Poisson’s ratio
C2	403.10	0.30
C3	589.43	
C4	1,027.46	
C5	914.93	
C6	694.04	
C7	527.53	
T1	275.07	
T2	352.26	
Nucleus pulposus	1.0	0.49
Anulus fibrosus	3.4	0.40

The intervertebral disc was added to the space between the adjacent vertebral bodies, which was divided into the annulus fibrosus and nucleus pulposus. The structure of the nucleus pulposus covered 25%–50% of the surface of the upper and lower vertebral bodies and 40%–50% of the volume of the entire intervertebral disc (Iatridis et al., 1996; Oegema, 1993; Pooni et al., 1986). The reconstructed intervertebral disc was attached to the upper and lower surfaces of the adjacent vertebral bodies, with all translational degrees of freedom constrained at the connection points. In order to reduce the nonlinearity of the finite element model, the linear-elastic mechanical properties of the core and ring were determined. To simplify the model calculations, the fibers inside different layers of the intervertebral disc have not been considered.

Six groups of ligaments, posterior longitudinal ligament (PLL), anterior longitudinal ligament (ALL), interspinous ligament (ISL), supraspinous ligament (SSL), intertransverse ligament (ITL) and ligamentum flavum (LF) were developed using tension-only rod elements and attached to the corresponding vertebrae (Table 2). Only one element was mapped for each filament in every ligament (T3D2), where only tension loads are active and there is no moment transfer.

**TABLE 2:** Mechanical properties and cross-sectional area of each ligament.

Ligament	Young’s modulus (MPa)	Poisson’s ratio	Cross-section area (mm^2^)
Posterior longitudinal ligament (PLL)	70	0.3	20
Anterior longitudinal ligament (ALL)	20		38
Interspinal ligament (ISL)	28		35.5
Supraspinal ligament (SSL)	28		35.5
Intertransverse ligament (ITL)	50		10
Ligamentum flavum (LF)	50		60

### 2.2 Biomechanical testing

The FE model of intact C2–T2 segments was fixed at the inferior endplate of T2. The patient’s head weight was estimated to be 61.4 N based on the average head weight of 7.83% of body weight reported in the literature (Ramachandran et al., 2016).

**FIGURE 1:**
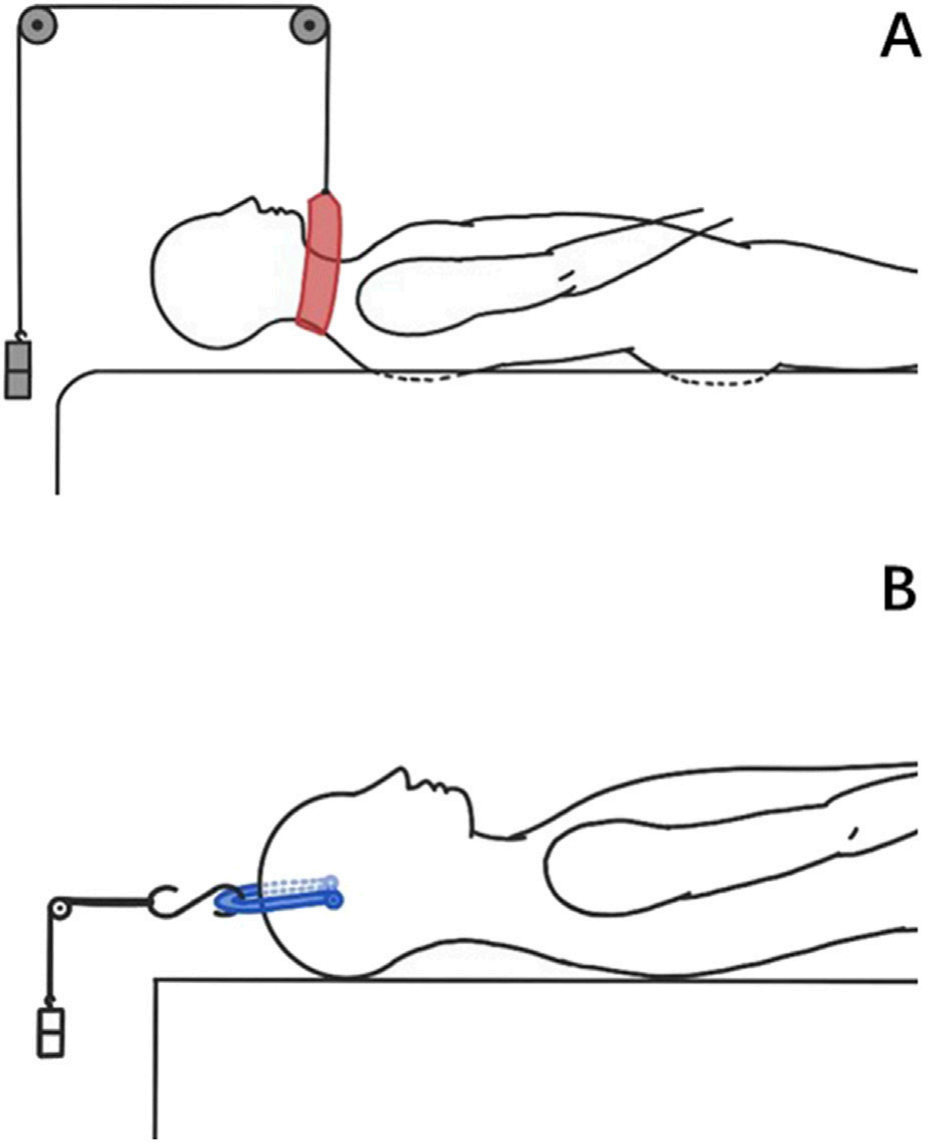
Schematic diagram of traction. **(A)** Suspensory traction. **(B)** Axial traction.

The protocols for axial traction and suspension traction have been reported in the literature (Shen et al., 2019; Wu et al., 2012), with differences in the direction and magnitude of the traction force (Figure 1). In axial traction, a traction load of 15 kg is applied by multi-point control. A reference point is created behind the cervical spine, and all degrees of freedom of the vertebral mesh nodes and the reference point are bound, which means the degrees of freedom of motion of the vertebral mesh nodes and the reference point are consistent. The gravity of the head is applied to C2 in the same way, perpendicular to the axis of the cervical spine, to simulate the state of skull traction in the supine position. The additional bending moment caused by the change of the force point is also applied to C2. In suspensory traction, a traction force of 10 kg was evenly applied to the C3 spinous process to simulate the force provided by the traction harness. The gravity of the head is applied in the same way as in axial traction. According to radiographic measurements, the arm of force of head gravity is 65 mm.

The kyphotic Cobb angle was defined as the angle between the inferior endplates of the uppermost and lowermost vertebral bodies in the cervical kyphotic segments, which in this patient is C2-C5. The length of the cervical spinal canal was measured with reference to the previous method of measuring the length of the lumbar spinal canal by CT (Yahara et al., 2019). The center point of each segment of the spinal canal was determined by the center point of the pedicle and the endplate. The distances between each point were added to obtain the length of the spinal canal. Additionally, the maximum von Mises stress on each cervical vertebral body under the two traction conditions was calculated.

## 3 Results

### 3.1 Kyphotic cobb angle

**FIGURE 2:**
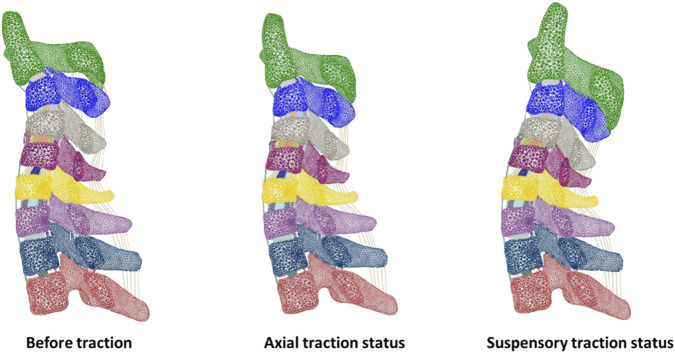
Sagittal sections of the finite element model of the cervical spine before traction and under two traction modes.

The preoperative kyphotic Cobb angle was 41°, and the condition of kyphosis improved after both two kinds of tractions (Figure 2). In axial traction, the kyphotic Cobb angle dropped to 32°, which was 26° in suspensory traction.

### 3.2 Length of the cervical spinal canal

The length of C3-7 spinal canal before traction was 61.3 mm. After axial traction, its length increased to 61.8 mm. In contrast, its length dropped to 59.6 mm after suspensory traction (Table 3).

**TABLE 3:** Length of cervical spinal canal in three different states.

Segment	Before traction (mm)	Axial traction status (mm)	Suspensory traction status (mm)
C3-4	16.2	14.8	15.5
C4-5	16.3	14.9	15.1
C5-6	14.9	16.7	14.9
C6-7	13.9	15.4	14.1
C3-7	61.3	61.8	59.6

### 3.3 Stress of vertebral body

**FIGURE 3:**
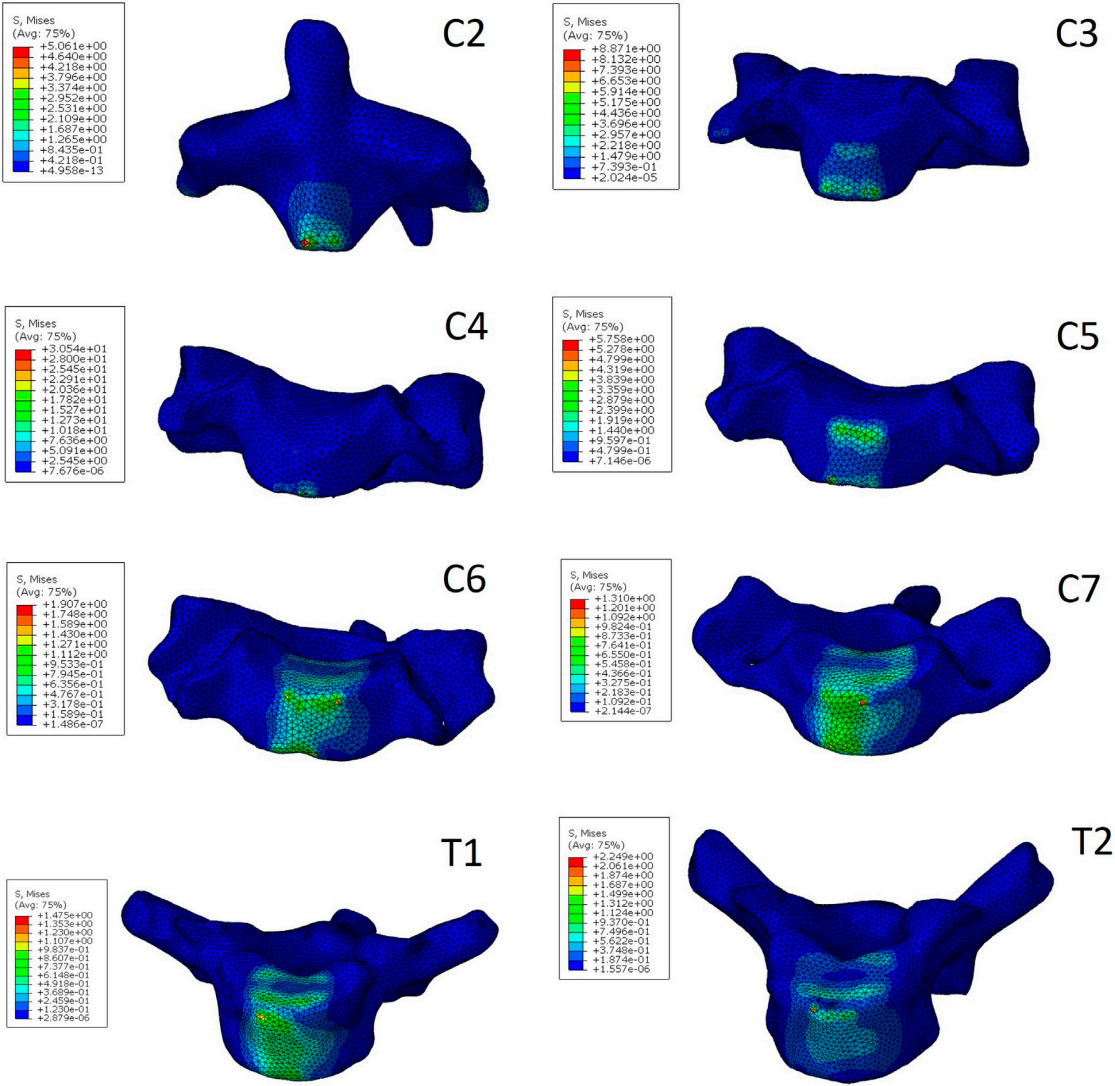
The von Mises stress distribution of C2-C7 vertebrae in axial traction.

**FIGURE 4:**
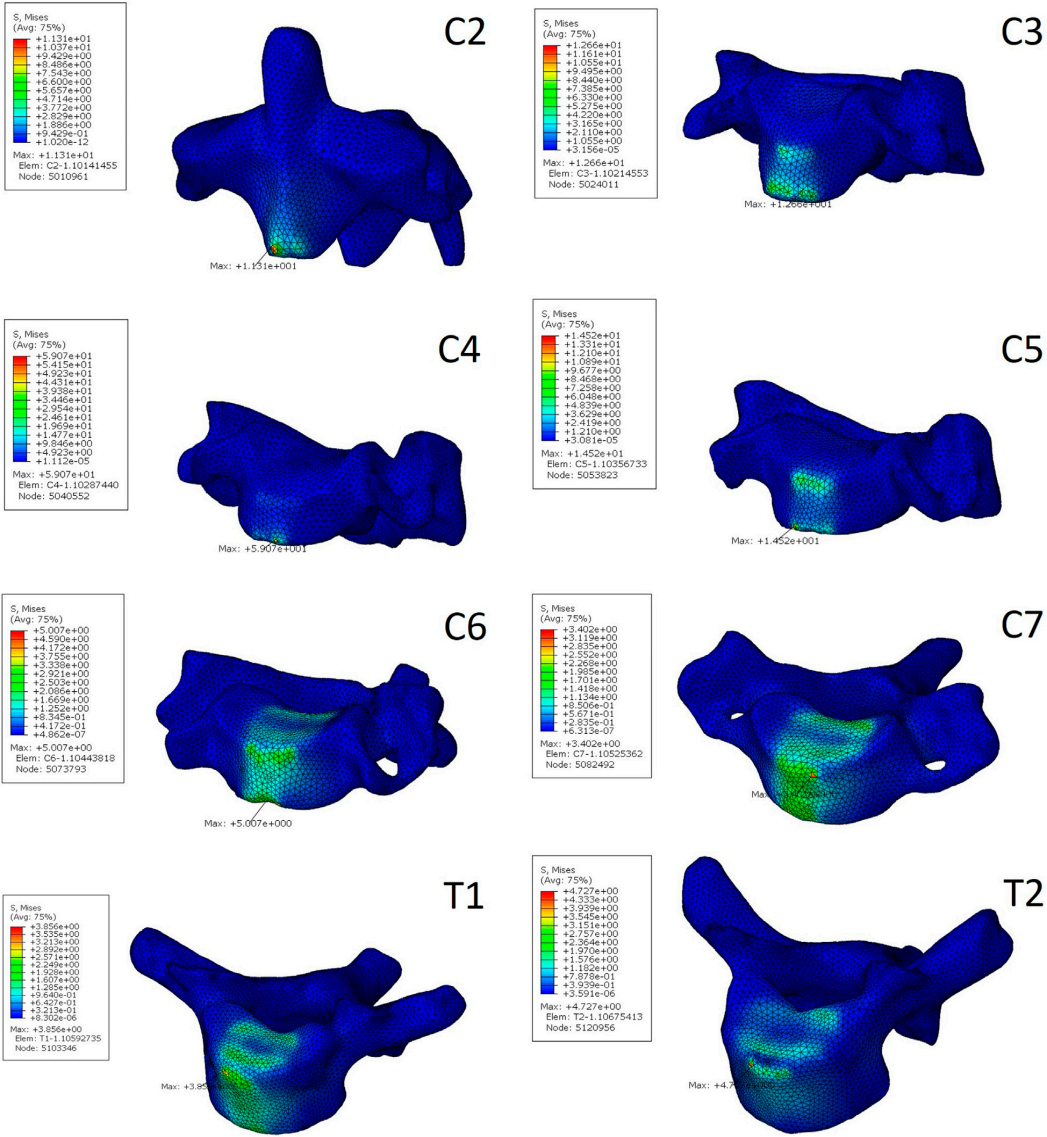
The von Mises stress distribution of C2-C7 vertebrae in suspensory traction.

**FIGURE 5:**
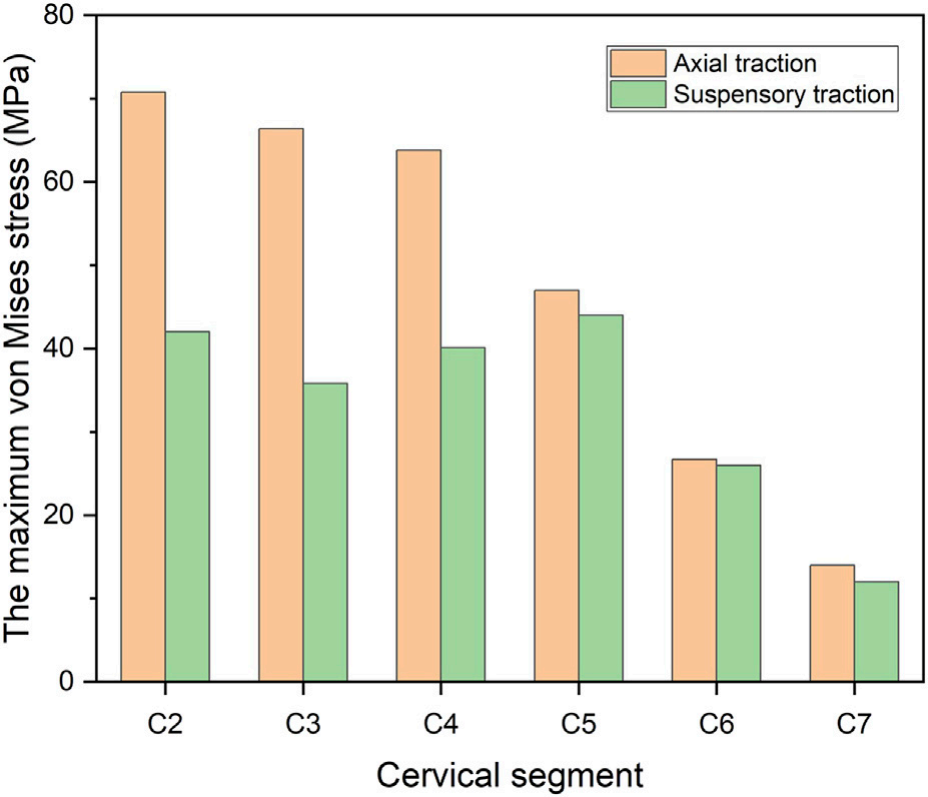
The maximum von Mises stress of each cervical segment under two traction modes.

The stress distribution characteristics of the two traction states were similar, and the von Mises stress concentration area was located in the anterior area of the vertebral body (Figures 3, 4), which is the attachment point of the anterior longitudinal ligament. In suspensory traction, the maximum von Mises stress of C2-C7 vertebral body was 70.8 MPa, 66.4 MPa, 63.8 MPa, 47.0 MPa, 26.7 MPa and 14.0 MPa respectively. In axial traction, the maximum von Mises stress of C2-C7 vertebral body was relatively small, which was 42.0 MPa, 35.8 MPa, 40.1 MPa, 44.0 MPa, 26.0 MPa and 12.0 MPa respectively (Figure 5).

## 4 Discussion

Traction before cervical kyphosis correction surgery can slowly and safely release part of the tension caused by soft tissue, significantly improve the correction effect and safety of the operation (Goffin and Grob, 1999; Horsley et al., 1997; Iwasaki et al., 2002). Therefore, it has become a general consensus for the treatment of severe cervical kyphosis that traction should be performed before surgical decompression and fixation. However, the traditional axial traction have drawbacks. It has been reported that the nerve injury is aggravated due to improper traction (Goffin and Grob, 1999), which is caused by the stretching of the whole length of the cervical spine and the corresponding traction of the spinal cord nerve roots. In addition, the stress of axial traction may also cause iatrogenic fractures, especially in patients with osteoporosis and anterior soft tissue tension.

In order to improve and solve these drawbacks, the authors have used cervical suspensory traction in recent years, and achieved good clinical results (Shengfa et al., 2023). A vertical upward traction force is generated through the neck traction belt. The vertex of the cervical vertebra kyphosis is pressed, and a rotating traction force to the back of the neck is generated depending on the weight of the head by taking the zygapophysial joint as rotating shaft, which is different from the direction of a traditional axial traction. Suspensory traction allows the contracted anterior longitudinal ligament, longus colli and anterior annulus fibrosus of the intervertebral disc to be retracted, while ensuring the structures behind and on both sides of the cervical spine are not affected, which can slowly and safely release part of the tension caused by soft tissues. The results of the study also demonstrate that the suspensory traction exerts less traction on the cervical spine than axial traction, while produces better kyphotic correction.

At present, there is no effective method to measure the tension of spinal cord directly. Some scholars indirectly reflect the tension of spinal cord by measuring the length of spinal cord or spinal canal (Mahesh et al., 2016; Yahara et al., 2019). Kuwazawa et al. (2006). And Zhang et al. (2011) demonstrated that the spinal cord in flexion was longer than that in neutral or extension by taking MRI in different positions in healthy volunteers and patients with cervical spondylotic myelopathy, respectively (Mahesh et al., 2016). found a positive correlation between the shortening of the spinal canal and clinical improvement after anterior surgical correction of cervical kyphosis, which he attributed to the reduction of spinal cord tension. In the present study, the tension of the spinal cord in different states was evaluated by measuring the length of the cervical spinal canal in the finite element model. The length of C3-7 cervical spinal canal in suspensory traction decreased by 1.7 mm compared with that before traction, which is because the kyphotic spinal canal was straightened with the posterior cervical spine as the rotation axis during traction. The increase in the length of the cervical spinal canal during axial traction was not significant compared with that before traction, which was presumed to be due to the lengthening of the cervical spine caused by traction during axial traction was offset by the decrease in the length of the spinal canal caused by kyphosis correction. Compared with axial traction, the length of cervical spinal canal is shorter in suspensory traction, which has potential advantages in improving neurological function and avoiding neurological damage.

This study also analyzed the stress distribution of each segment of the vertebral body under the two kinds of traction modes. It was found that the stress caused by the two kinds of traction both concentrated on the anterior part of the vertebral body, which is the attachment area of the anterior longitudinal ligament. This kind of stress distribution is beneficial to the improvement of cervical kyphosis, but excessive stress concentration will cause damage to the vertebral body. The risk of iatrogenic fracture is higher in patients with NF-1 and osteoporosis (Lim et al., 2020; Mladenov et al., 2020). In the present study, it was found that the maximum von Mises stress of cervical vertebral body under suspensory traction was relatively smaller, so it can be considered that it is safer in the preoperative correction process for patients with cervical kyphosis with poor bone quality. In axial traction, the maximum von Mises stress on the vertebral body decreased gradually away from the head, but this trend was not reflected in the suspensory traction state. Under both two kinds of traction, the lower cervical vertebra is subject to less tension, so iatrogenic fractures are more likely to occur in the upper cervical vertebra during preoperative correction traction of kyphosis, which is consistent with clinical observations (Lim et al., 2020).

Finite element analysis is an important means to study the biomechanical environment of cervical spine under different conditions, so as to provide some guidance for clinical treatment (Hua et al., 2020; Wu et al., 2019). However, several limitations of the current study should be considered. First, the kyphotic segments of the selected patient were located at C2-C5. However, cervical kyphosis occurs in a variety of regions, so the results may not cover all cases of cervical kyphosis. Second, the model lacks the corresponding creep material composition of soft tissue such as neck muscles, and can not simulate the process of contracted soft tissue being retracted during preoperative traction of cervical kyphosis, which may make the load-sharing behavior of the model different from that of the actual situation. Third, although a finite element model of kyphosis is constructed, the material properties assigned to ligaments and discs are data from healthy population, so the model may not be a perfect representation of a real clinical scenario.

## 5 Conclusion

The results of finite element analysis showed that compared with traditional axial traction, suspensory traction had the advantages of greater correction degree, lower possibility of nerve traction injury and iatrogenic fracture for preoperative correction of cervical kyphosis. Therefore, suspensory traction can be used as an important traction method in the staging treatment of cervical kyphosis.

## Data Availability

The raw data supporting the conclusions of this article will be made available by the authors, without undue reservation.
